# A Practical Treatise on Foreign Bodies in the Air Passages

**Published:** 1855-07

**Authors:** 


A Practical Treatise on Foreign Bodies in the Air Passages. By S. D. Gross, M. D., Prof. of Surgery in the University of Louisville, etc., etc., with illustrations. Philadelphia: Blanchard & Lea. 1854.

The work of Dr. Gross is a valuable contribution to statistical surgery. The author has carefully analyzed the cases that have been reported from time to time in the public journals, and in addition has reported about fifty original cases. Bronchotomy is recommended as the only reliable means of removing the foreign body. This should be resorted to as early as possible, unless contra-indicated by special conditions. We have no doubt in our own mind but that this operation often fails from having been delayed until the inflammation of the air passages has become too extensive to admit of recovery. We think this is the reason why tracheotomy for croup has so often been followed by unfavorable results. The work is valuable for reference, and is well worthy of a place in the library of every physician and surgeon. J.

